# Geographic Structuring of the Tomato Brown Rugose Fruit Virus Populations in Morocco

**DOI:** 10.3390/v18091032

**Published:** 2026-09-17

**Authors:** Ayoub Maachi, Livia Donaire, Miguel A. Aranda

**Affiliations:** 1 Regional Center of Agricultural Research, National Institute of Agricultural Research (INRA), Inezgane, Agadir 86350, Morocco; 2 Department of Stress Biology and Plant Pathology, Centro de Edafología y Biología Aplicada del Segura (CEBAS)-CSIC, Espinardo, 30100 Murcia, Spain; ldonaire@cebas.csic.es (L.D.); m.aranda@cebas.csic.es (M.A.A.)

**Keywords:** ToBRFV, genetic variability, haplotype network, tomato fruits, movement protein

## Abstract

Tomato brown rugose fruit virus (*Tobamovirus fructirugosum*, ToBRFV) is an emerging virus that affects tomatoes, capsicum, and chili. Since its first detection in Jordan in 2015, the virus has been reported in more than 40 countries across all the continents. In Morocco, the virus was reported for the first time in October 2021. However, its genetic diversity remains unexplored. In this work, we used a collection of 100 tomato fruits from local markets to investigate the virus’s variability. Thirty-eight sequences were recovered and used to study evolutionary pressures acting on the N-terminus of the RNA-dependent RNA polymerase, the movement protein (MP), and the coat protein (CP) genes. The genetic diversity among Moroccan sequences was low, with over 99% nucleotide identity, which is consistent with the global situation, with the CP exhibiting higher diversity followed by the MP. We identified two sites with non-synonymous substitutions in the CP, and one in the MP. We used haplotype network analyses to reveal the population structure within the Moroccan isolates and studied their relationships with sequences from the rest of the world. Sequences from Morocco showed a clear geographic structure, suggesting that geographic factors, potentially combined with agricultural practices, may contribute to shaping the population structure of ToBRFV in Morocco. Our analyses suggest few introduction events, probably from Israel, Jordan, and Italy.

## 1. Introduction

Tomato (*Solanum lycopersicum* L.) is one of the most important vegetable crops in Morocco, with an estimated production of 1,686,615 tons in 2024 (FAOSTAT; http://www.fao.org/faostat/; accessed in March 2026). The crop generates $1.15 billion in revenue through trade with the EU and other countries, contributing to 1% of Morocco’s overall Gross Domestic Product (GDP). Tomato cultivation is very intensive and is concentrated mainly in the Souss-Massa region, with significant acreage devoted exclusively to greenhouse production. Viral diseases are considered major threats to intensive tomato cultivation, which are responsible for significant yield and fruit quality losses [1]. The main viruses frequently reported to affect tomatoes are tomato yellow leaf curl virus (*Begomovirus coheni*) and pepino mosaic virus (*Potexvirus pepini*) [2]. Nevertheless, emerging viruses, i.e., those that have recently appeared in a population for the first time, or that have existed previously but are rapidly increasing in incidence or geographical range, often cause the most important problems. A recent example of an emerging virus infecting tomato crops is tomato brown rugose fruit virus (ToBRFV) (*Tobamovirus fructirugosum*; family *Virgaviridae*).

ToBRFV is an emerging tobamovirus that affects tomato, capsicum (*Capsicum annuum*), and chili (*Capsicum frutescens*) [3,4,5]. In tomato and depending on the variety, ToBRFV may cause leaf chlorosis, mosaic patterns, and mottling on younger leaves. On the fruits, the virus may cause chlorotic spots and marbling, deformation and uneven ripening, and brown rugose patches [5]. ToBRFV is a stable and highly infectious virus that is easily transmitted by mechanical means, via seeds [5,6], bumblebees [7], and contaminated water [8] and soil [9], enabling its spread locally and over long distances. The virus can persist for a long period of time on different structures at production sites [10,11]. Since its first report in Jordan in 2015, the virus has been reported in more than 40 countries across all the continents. ToBRFV showed exponential population growth until 2020, followed by a decline in estimated effective population size, likely due to control measures or saturation [12]. The variability of ToBRFV is limited, with countries experiencing single or multiple introduction events [12]. In Morocco, ToBRFV was first reported in October 2021 in the Souss-Massa region (EPPO Reporting Service: 2023/2025).

In this study, we used a collection of tomato fruits produced in Morocco and gathered near the main production farms in the Souss-Massa region to detect ToBRFV’s presence and study its diversity and the evolution of local isolates vis-a-vis the worldwide population, attempting to determine possible introduction pathways of the virus into the country.

## 2. Materials and Methods

### 2.1. Sample Collection and Nucleic Acid Extraction

**Figure 1:**
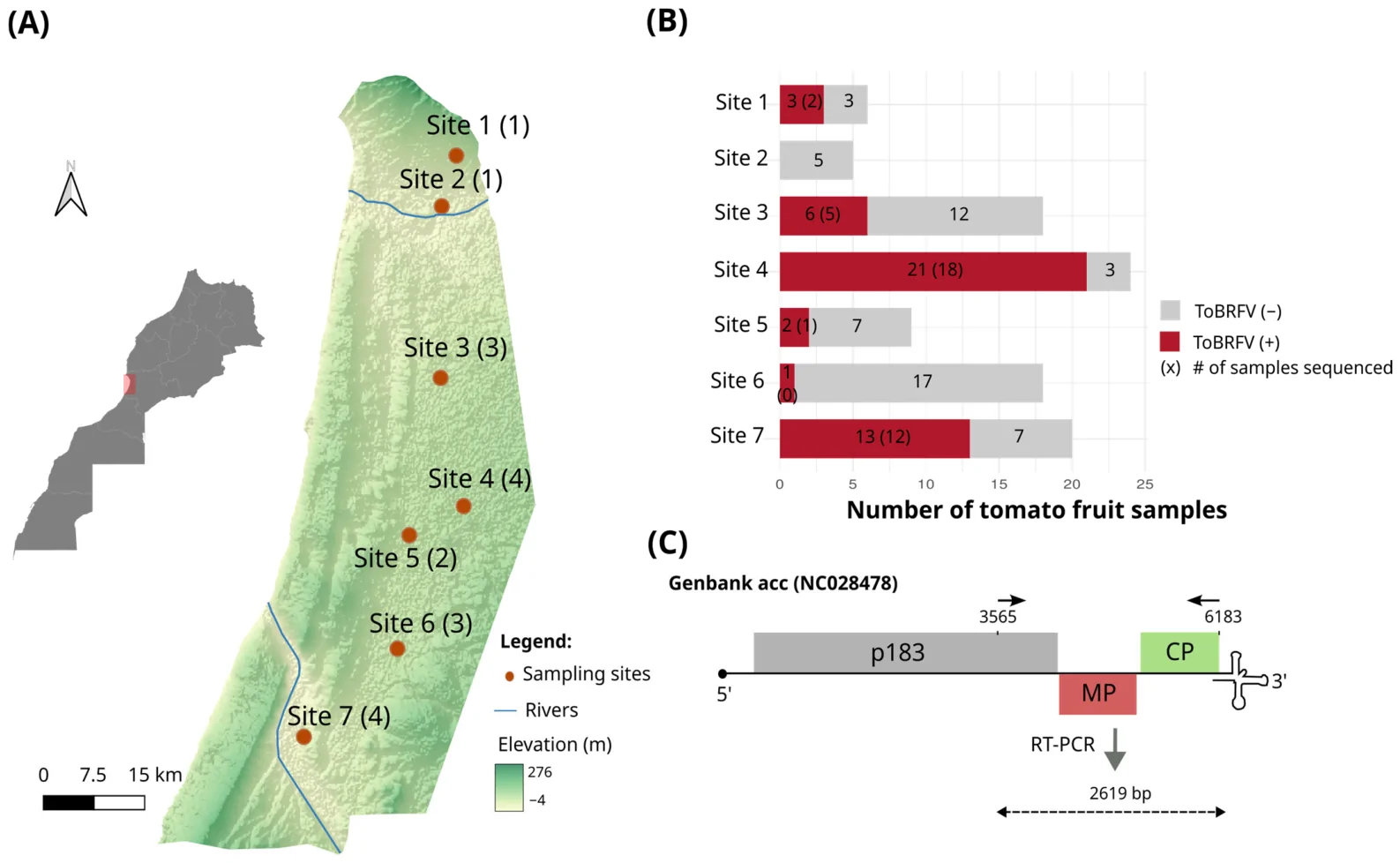
Collection sites and tomato brown rugose fruit virus (ToBRFV) detection from different vegetable markets. (**A**) Map showing the location of the different markets visited for sample collection. The figures in brackets indicate the number of shops visited per market. (**B**) Number of tomato fruit samples collected from each site, and the proportion of ToBRFV-infected fruits from each market. The horizontal bars in red indicate the number of samples positive for ToBRFV, while the gray ones indicate negative samples. The figures in brackets indicate the number of positive samples sequenced for genetic diversity analyses. (**C**) Diagram representing the ToBRFV genome. The arrows indicate the position of the primers and the region used for virus detection and genetic diversity analyses in this study.

One hundred tomato fruit samples exhibiting uneven ripening and symptoms compatible with ToBRFV infection were collected from shops in markets located near the main tomato-producing areas in the Souss-Massa region, except for two sites which were located inside the city of Agadir. Samples were collected from seven different market sites during the period from May to June 2025. In each market, one to three shops were visited (Figure 1A), and four to eight samples were collected from each shop. Total RNA was extracted using the RNA preparation method from Souiri et al., with modifications [13]. Briefly, 100 mg of pulp, skin and juice of tomato was ground with 100 μL of sterile distilled water in a 1.5 mL microtube using cotton swabs; then, total volume was made up to 1 mL with sterile distilled water. After precipitation for 1 h at 4 °C, the supernatant was recovered in a new microtube, and RNA was precipitated using 0.1 volume of 3M NaOC (pH 5.2) and 2 volumes of absolute ethanol. The pellet was washed using 1 mL of 70% ethanol and kept at 4 °C for downstream analysis.

### 2.2. ToBRFV Detection and Amplicon Sequencing

ToBRFV was detected in individual fruit samples by RT-PCR using primers LD32F: 5′-TGTTACCATGAGGTTGACTGAC-3′ and LD35R: 5′-GGTGCAGAGGACCATTGTAA-3′ designed for this study (Figure 1C). The specificity of the primers was confirmed in silico by a blastn search against the National Center for Biotechnology Information (NCBI) database. Complementary DNA was synthesized with SuperScript III reverse transcriptase (Thermo Fisher Scientific, Waltham, MA, USA) using the primer LD35R. Each 20 μL reaction consisted of 0.2 µL of 10 µM LD35R, 1 µL of 10 mM dNTP mix, 4 µL of 5× First Strand Buffer, 1 µL of 0.1M DTT, 1 µL of RNase inhibitor, 1 µL of SuperScript III, 8.8 µL of sterile distilled water and 3 µL of RNA. PCR reactions were carried out in a final volume of 25 µL. Each reaction consisted of 12.5 µL of CloneAmp HiFi PCR Premix (Takara Bio, Kusatsu, Japan), 0.5 µL of LD32F/LD35R (10 µM), 2 µL of cDNA, and 9.5 µL of sterile distilled water. The reactions were incubated in the thermocycler under the following conditions: 98 °C for 2 min, 35 cycles (98 °C for 10 s, 55 °C for 10 s, and 72 °C for 2 min) and 72 °C for 3 min. The PCR products with the expected size (≈2619 bp) were purified using the NZY Gelpure kit (NZYtech, Lisboa, Portugal) and subjected to amplicon-free Oxford Nanopore sequencing by Eurofins Genomics. Libraries were prepared using Oxford Nanopore Technologies V14 chemistry with minimal fragmentation and sequenced on a GridION platform using R10.4.1 flow cells. Raw reads were processed and assembled using the Eurofins Genomic Amplicon Analysis Pipeline v3.1.1 to generate consensus amplicon sequences. Sequences with low quality were removed, and the remaining ones were curated manually using GeneiousPrime 2024.0.7, yielding 38 consensus sequences, obtained from fruits collected at the same 7 sites depicted in Figure 1, with a length of 2589 bp for the subsequent analyses.

The generated sequences were complemented using 11 additional ToBRFV sequences from GenBank at the NCBI. These sequences were obtained from tomato fruits in the United Kingdom (UK) and the Netherlands (NL), but collected from Morocco between 2023 and 2025 (NCBI). The accession numbers of the complementary data are: PP099972 (NL, 2023), PV389033 (NL, 2024), PV389019 (NL, 2024), PV389020 (NL, 2024), PV389048 (NL, 2025), PV389021 (NL, 2024), and PV389041 (NL, 2024), PV389010 (NL, 2025), PP236969 (UK, 2023), PP236971 (UK, 2023), and PP236970 (UK, 2025).

### 2.3. Genetic Diversity, SNP Calling and Selection Pressure Analyses

Multiple sequence alignments were performed using MUSCLE v5 with the -super5 option [14], followed by manual curation. DnaSP6 software was used to calculate the nucleotide diversity (π) in the whole fragment and in individual genes (partial RNA-dependent RNA “RdRp”, movement protein “MP”, and coat protein “CP”) using the Jukes and Cantor method.

Genetic variability was assessed using Shannon entropy. For each alignment position, the Shannon entropy (H) is calculated based on the frequency of each nucleotide, using the formula *H* = −*Σx_i_log*_2_*x_i_*, where x_i_ represents the frequency of nucleotide (i) at a given position.

SNP calling was performed on GeneiousPrime, by mapping each of the genes obtained from assembled consensus sequences to the ToBRFV reference sequence under the accession number NC028478 and using the “Find Variations/SNPs” function with the minimum variant frequency set to 0.05.

Selective pressures acting on entire genes were determined using BUSTED (Branch-site Unrestricted Statistical Test for Episodic Diversification), while selective pressures acting on individual codons were determined using FEL (Fixed Effects Likelihood), which uses a maximum-likelihood approach to infer non-synonymous (dN) and synonymous (dS) substitution rates on a per-site basis. Both algorithms were run on the Datamonkey web server (datamonkey.org).

### 2.4. Haplotype Network Reconstruction and Phylogenetic Analyses

Haplotype networks (HNs) were constructed using the PopART 1.7 software using the median-joining haplotype network, which is a common method to depict relationships between closely related sequences of the same species or population [15].

A HN was built using only sequences from Morocco. Another HN was generated by combining ToBRFV sequences from Morocco and the rest of the world. A total of 473 sequences of ToBRFV, longer than 6148 nts, were downloaded from the NCBI Virus database. Sequences were mapped to a ToBRFV (1) sequence (PZ370457, generated in this study) and trimmed to comprise the RdRp, the MP, and the CP. Two hundred and seventy unique sequences were kept for the analyses using the Seqkit tool. These sequences were merged with the ones generated from this study and were used for the analyses.

### 2.5. Structural Prediction and Residue Analysis of the MP

Five hundred twenty-seven MP sequences were retrieved from the NCBI Virus database (https://www.ncbi.nlm.nih.gov/labs/virus/vssi/#/ (accessed on 1 April 2026)) using the following Virus/Taxonomy: “tomato brown rugose fruit virus; taxid 1761477”. Incomplete sequences were removed, and the remaining ones were added to the sequences generated from this study. The SeqKit tool was used to remove redundant sequences, yielding 69 unique sequences. Genetic variability within the MP was determined using Shannon entropy and ConSurf [16].

Protein structure of the MP (accession number: WEX498921.1) was predicted using AlphaFold3 [17] via the online server (https://alphafoldserver.com/ (accessed on 2 April 2026)). Predicted structure was converted from CIF to PDB format using the mCIF-to-PDB converter tool (https://project-gemmi.github.io/wasm/convert/cif2pdb.html (accessed on 2 April 2026)). Specific sites were annotated on the 3D structure using PyMOL 3.1 (https://www.pymol.org/ (accessed on 2 April 2026)).

## 3. Results

### 3.1. ToBRFV Detection in Tomato Fruit Samples

We sampled 100 tomato fruits from eighteen shops located in seven different local markets within the Souss-Massa region (Figure 1A). The visited markets were located near the tomato cultivation zones. Among the samples collected, molecular detection by RT-PCR revealed ToBRFV infection in 50 samples. Positive samples ranged from one fruit at Site 6 to twenty-one fruits at Site 4 (Figure 1B). No sample from Site 2 was positive (Figure 1B).

### 3.2. Genetic Diversity and Selective Pressures Among ToBRFV Sequences

**Figure 2:**
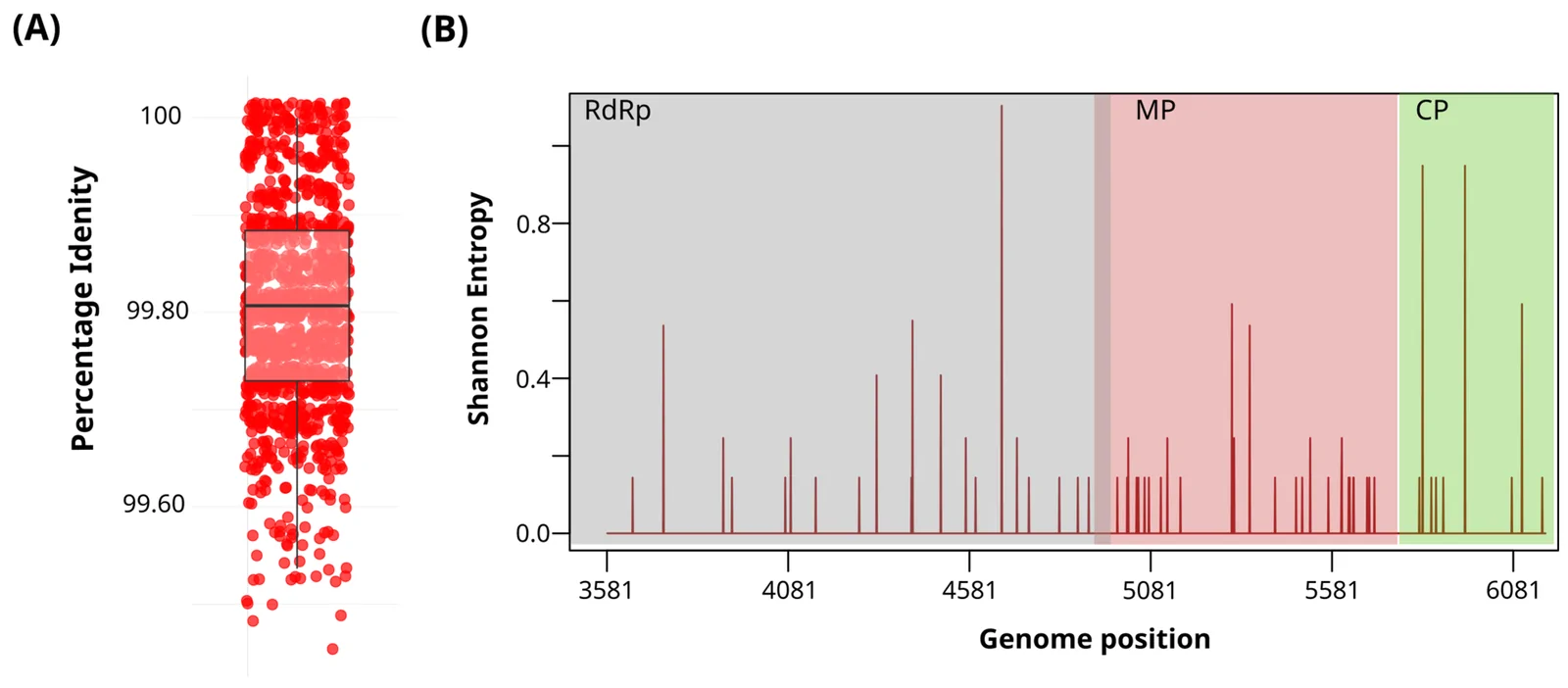
Genetic variability of tomato brown rugose fruit virus (ToBRFV) sequences from Morocco. (**A**) Percentage of pairwise nucleotide identity among the different sequences from Morocco. (**B**) Genetic variability across the N-terminus region of the RNA-dependent RNA polymerase (RdRp), the movement protein (MP), and the coat protein (CP) genes of ToBRFV using Shannon entropy.

We sequenced a genomic region comprising ≈37% (2589 bp in length) of the ToBRFV genome covering the partial RdRp ORF (1344 bp), the MP, and the CP ORFs (Figure 1C). We sequenced this segment from 38 samples, yielding one sequence per infected fruit. Amplicon sequencing yielded amplicons with lengths ranging from 2558 nt to 2589 nt, assembled from total reads ranging from 1684 reads to 11,819 reads per sample (Figure S1), with a coverage depth ranging from 28× to 2774× per sample (Figure S1). We complemented our data with 11 additional sequences of ToBRFV from tomato fruits from Morocco; these were previously determined and made publicly available in the GenBank database. Pairwise nucleotide identity among the sequences ranged from 99.45% to 100% (Figure 2A). Overall, the nucleotide diversity was low (π = 1.91 × 10^−3^ ± 1.7 × 10^−4^), and diversity was unevenly distributed across the sequenced fragment (Figure 2B), with RdRp showing the lowest diversity (π = 1.45 × 10^−3^ ± 1.8 × 10^−4^), followed by the MP (π = 1.89 × 10^−3^ ± 10^−7^) and the CP, which exhibited the highest diversity (π = 3.08 × 10^−3^ ± 10^−7^).

We next investigated the selective pressures acting on each of these genes. No evidence of episodic diversifying selection was detected in entire genes, nor in single codons (Table S1–S3). However, we identified sites with non-synonymous substitutions. A guanine was substituted by adenine at site 5304 of the genome within the MP gene with a variant frequency of 14.3% (*p* < 0.001), changing the codon from GAA to AAA, leading to the putative change of the amino acid from glutamic acid (negatively charged) to lysine (positively charged). Two non-synonymous substitutions were identified in the CP gene, one in position 5947 substituting a thymine into adenine with a variant frequency of 36.7%, resulting in the amino acid changing from aspartic acid (codon GAU) to glutamic acid (codon GAA) (*p* < 0.001). Another mutation was detected in position 6104 substituting the guanine into adenine with a variant frequency of 14.3%, resulting in the amino acid changing from valine (codon GUA) to isoleucine (codon AUA) (*p* < 0.001).

### 3.3. Relationship Among the ToBRFV Sequences in Morocco and the Rest of the World

**Figure 3:**
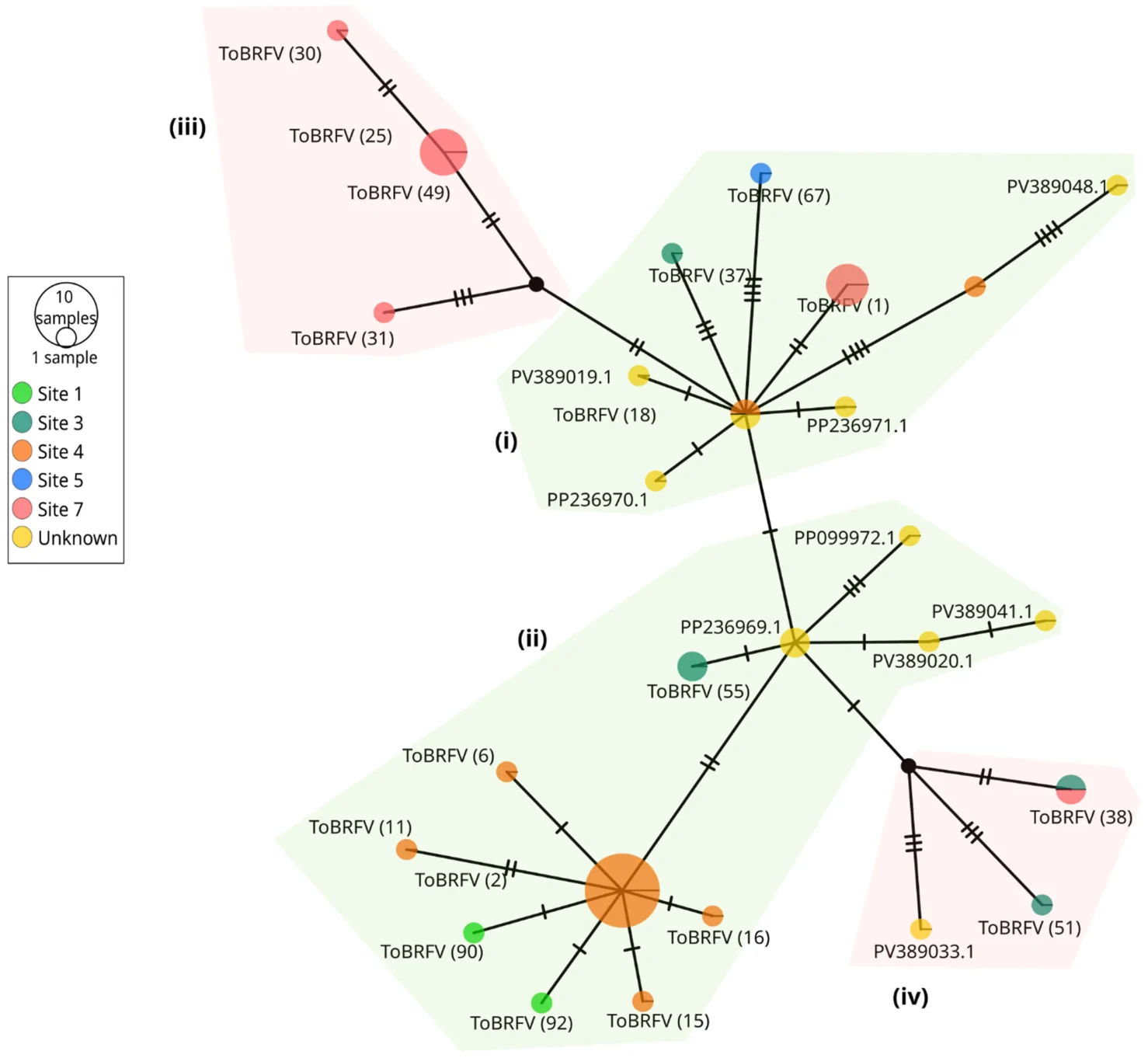
Relationships among the tomato brown rugose fruit virus (ToBRFV) sequences from Morocco using “median-joining” haplotype networks. Different clusters or groups are highlighted in light green and light red. The Arabic numbers between parentheses indicate the fruit ID from which the virus sequence was recovered (i.e., ToBRFV (25) means ToBRFV sequence recovered from tomato fruit number 25). (i)–(iv) indicates ToBRFV subpopulation number.

We explored the relationship between the different ToBRFV sequences using haplotype network construction. Our results revealed 27 haplotypes clustered into four main groups (i to iv in Figure 3). Groups (i) and (ii) are central in the network, while groups (iii) and (iv) are located on the edges of the network. Groups (i) and (ii) exhibit predominant haplotypes connected to several variants by one to four mutations, while groups (iii) and (iv) are separated from the main clusters by intermediate haplotypes (Figure 3).

The distribution of haplotypes across the different sites showed that several haplotypes were shared among the different locations, although no sensu stricto clustering was observed by site. However, some subclusters were composed predominantly of samples from the same site, suggesting partial structuring within the population (Figure 3).

**Figure 4:**
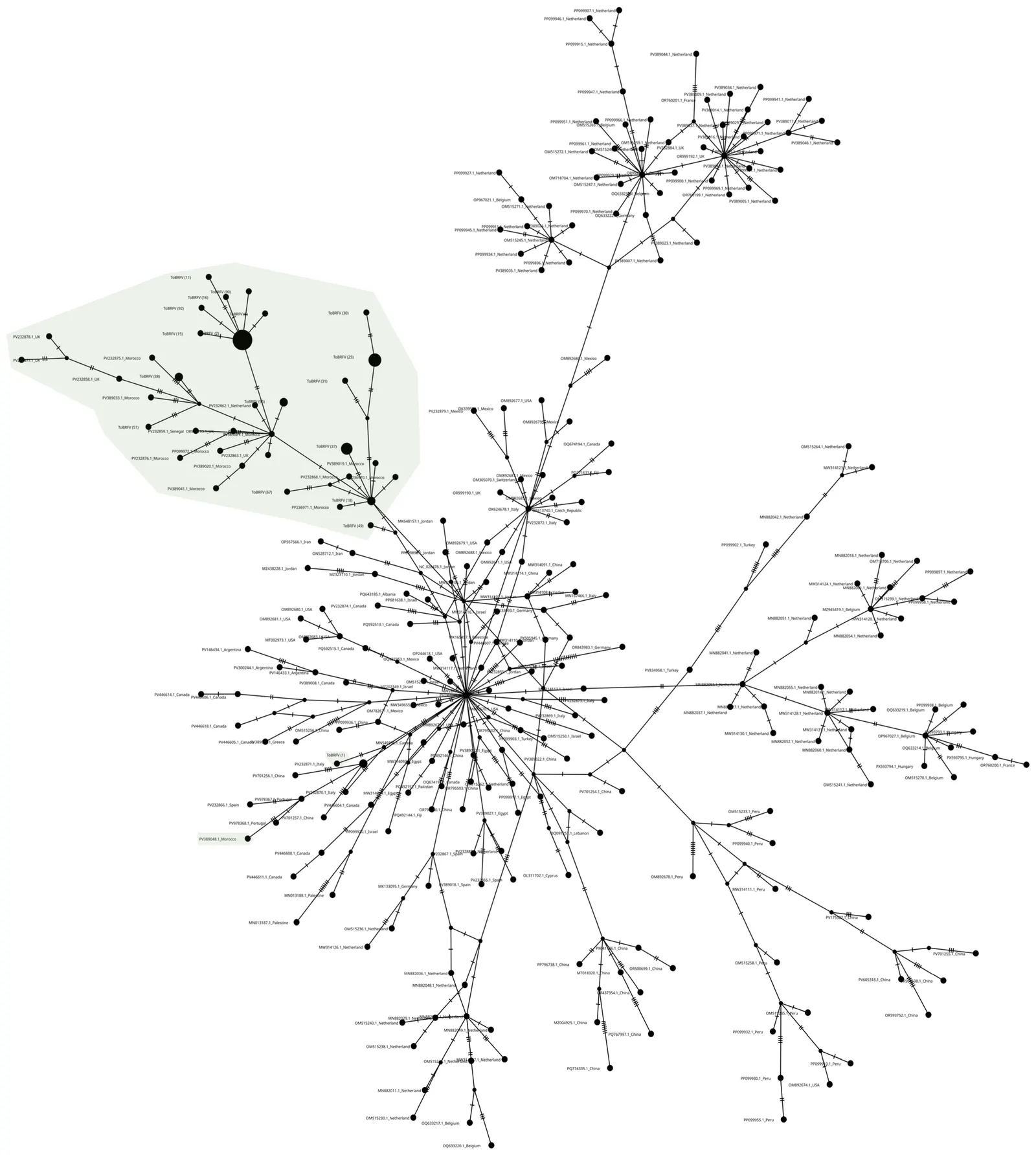
Relationships among the tomato brown rugose fruit virus (ToBRFV) sequences from Morocco and the rest of the world using “median-joining” haplotype networks. ToBRFV sequences from Morocco are highlighted in dark green. A network with higher resolution is provided in the Supplementary Material as Figure S2.

Next, we examined the relationships between ToBRFV sequences from Morocco and the rest of the world (Figure 4 and Figure S1). We observed clustering of the ToBRFV isolates into distinct clades. Sequences from Morocco were positioned in different clusters, although a major cluster comprised the majority of the sequences from Morocco. This cluster included sequences from Senegal, the Netherlands, and the UK. This cluster seems to have emerged from sequences from Jordan and Israel. Two additional sequences were clustered in a group comprising different isolates which does not seem to be well differentiated. This group comprises sequences from all the continents which were separated by a small number of mutations. We also noticed that some clusters comprised sequences from China, and others comprised sequences from the Netherlands. Sequences from the Netherlands appeared to be present in almost all the clusters. The overall network showed limited divergence among isolates, since haplotypes were connected by one to thirteen mutations, consistent with the low genetic variability of the virus. Clustering based on geographic origin was observed for the sequences from China, and for others from the Netherlands and Belgium; still, the ones from different countries were intermixed throughout the network.

### 3.4. Genetic Diversity Analyses of the Movement Protein of ToBRFV

**Figure 5:**
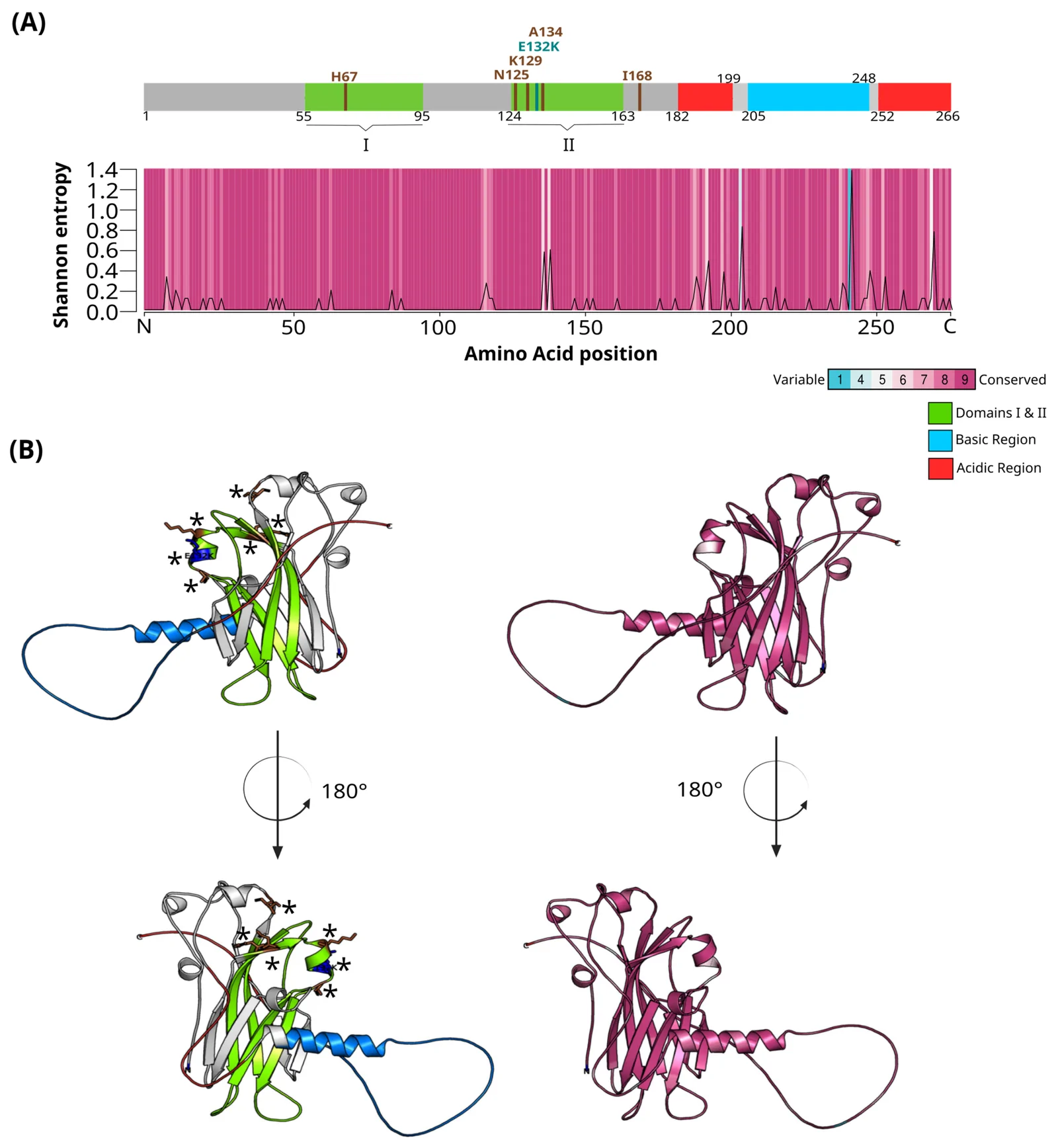
Genetic variability across the movement proteins (MPs) of tomato brown rugose fruit virus sequences using global sequences and ones generated in this study. Schematic representation of the different domains within the MP and the variability across them using the primary structure (**A**) and the quaternary structure (**B**). Important amino acids for the function of the MP are highlighted in brown in the primary and quaternary structures [19] The MP substitution reported from sequences in this study is highlighted in deep blue-green (teal) in the primary structure (3A) and in blue in the quaternary structure (3B). Asterisks were added to the quaternary structure to facilitate the visualization of the important residues. The light-blue to dark-purple colors indicate how an amino residue is conserved across the different movement proteins reported in ToBRFV worldwide; light blue indicates a variable amino acid residue while dark purple indicates a conserved residue, and this is for both the primary structure and the 3D structure. On the other hand, the colors green, blue, and red in the primary structure and 3D structure indicate specific domains reported from tobamovirus movement protein: green for domains I and II, blue for the basic region, and red for the acidic region.

Mutations in the MP help ToBRFV to evade *Tm*-2^2^ mediated resistance [18,19]. Therefore, we further studied the variability in the MP using globally available sequences (Figure 5). While this protein is well conserved, the variability observed was unevenly distributed across the protein (Figure 5A). The C-terminus, comprising the acidic and basic regions, exhibited higher variability than the N-terminus, which comprises the I and II domains. The E132K mutation observed in some ToBRFV sequences from Morocco was located in a conserved region within domain II, closer to N125, K129, and A134, which are critical residues for the function of the MP (Figure 5A,B).

## 4. Discussion

In this work, we used tomato fruits from local markets, produced in Moroccan farms, as sentinels to explore virus genetic diversity within farms in the Souss-Massa region. The use of sentinels as a sampling strategy combined with molecular tools was shown to be efficient in documenting the virosphere and disease emergence in different ecosystems and species [20].

The overall genetic diversity of the virus in Morocco is low, probably because of its recent introduction into the country, since official reports date back to 2021, or because of the strict control on seed importation imposed by the local phytosanitary authorities, limiting its introduction to the country. For this reason, we used haplotype network construction to depict relationships. The network revealed a structured population composed of 27 haplotypes clustered around two major nodes. These central haplotypes probably represent ancestral variants from which the current ToBRFV population in Morocco has diversified. The limited number of mutational steps connecting the haplotypes (one to four mutations) suggests relatively recent diversification. This pattern supports a scenario of population expansion, which is common for emerging viruses [21,22]. The presence of intermediate nodes may reflect unsampled haplotypes within the population or, alternatively, different independent introduction events of the virus to Morocco, although limited. Moreover, haplotype network analyses, using global sequences, showed a clustering of the Moroccan variants distinct from worldwide sequences, suggesting that geographical factors combined with agricultural practices may contribute to structuring the ToBRFV population from Morocco, playing a key role in shaping its evolutionary trajectory. A recent study from de Koning et al. [23] reported an increase in the incidence of a specific ToBRFV sequence in the EU. This sequence was only reported in the Netherlands and Belgium in 2021, whereas it has now been detected in four additional EU countries (France, Germany, Spain, and the UK) [23]. The rapid increase in the incidence of this sequence type is related to the deliberate and unauthorized application of a ToBRFV cross-protection product [23]. This sequence type was not among the Moroccan sequences, suggesting either its absence in Morocco or potential sample bias introduced by testing only symptomatic fruits.

The Moroccan cluster emerged from sequences from Jordan and Israel, suggesting a probable introduction from these countries. This cluster comprised sequences from the UK, Senegal, and the Netherlands, suggesting probable dissemination of the Moroccan variants via fruits. Generally, tomato fruits are not considered an important pathway for introductions and subsequent outbreaks of ToBRFV (EPPO, 2020; https://gd.eppo.int/taxon/TOBRFV/documents (accessed on 15 May 2026)). However, in both symptomatic and asymptomatic fruits, ToBRFV was found in relatively higher viral loads [24,25]. Our results corroborate recent findings from de Kooning et al., where introduction via fruits as a pathway was illustrated by a minor clade of sequences from Morocco and the UK [23].

The presence of two sequences, emerging from the ones from Italy, suggests a probable second route of introduction to the country. The low prevalence of these last variants may indicate their lack of competitiveness with the major ones observed in this study, or a lack of deep sampling to reveal their real prevalence across the country. Sequences from the Netherlands and Belgium were present in the different clusters, reflecting higher variability and probably suggesting multiple introductions of the virus to these countries and/or their role in its spread to other countries through the seed industry.

While previous studies have shown that the MP gene of ToBRFV exhibits the highest genetic variability at the global scale, suggesting its implication in virus evolutionary adaptation [26], our results show that the CP exhibited almost 1.7 times more diversity than the MP and the partial RdRp sequences. Despite CP variability, no episodic diversifying selection was identified, and the detected amino acid substitutions were conservative (D78E and V131I), suggesting that these variations are tolerated without strongly affecting function. These two mutations are located in two different alpha-helices, and 3D models do not support their belonging to shared functional CP sub-domains; however, they are in proximity in the quaternary structure of the CP, and therefore there is a possibility that they could act synergistically to alter binding affinity to host proteins, thereby changing viral functions including pathogenicity. Still, our results do not support that they are driven by adaptive diversification.

On the other hand, the MP substitution (E132K) involved a polarity change and may have greater functional relevance despite its lower frequency. The MP of tobamoviruses contains two highly hydrophobic regions that are predicted to span membranes [27]. The MP causes transient aggregation of the host’s endoplasmic reticulum, which apparently plays a role in the formation of cytoplasmic bodies where tobamoviruses replicate [28,29]. The MP is the primary determinant of resistance to tobamoviruses in hosts carrying the *Tm*-2^2^ resistance gene. ToBRFV was able to overcome this resistance present in the majority of commercial tomato cultivars. Mutations in the MP help ToBRFV to evade *Tm*-2^2^-mediated resistance by attenuating MP–*Tm*-2^2^ interaction strength, and/or Tm-2^2^’s level of expression [30]. While mutations in the MP can abolish cell-to-cell and systemic movement ([19]; Figure 3), a recent study showed that a single mutation in ToBRFV breaks virus-specific resistance in new resistant tomato cultivars [31]. The mutation was located within the MP substituting a (T) to (G), leading to amino acid change N82K in the MP of the resistance-breaking isolate [31]. We found that the MP of ToBRFV is highly conserved across the global sequences; still, the C-terminals comprising the acidic and basic regions are more variable. The mutation found in some of the Moroccan sequences corresponds to a conserved residue within domain II. This residue does not fall within the ones reported to inhibit MP function, possibly leading to adaptive diversification. Further biological assays should be carried out to assess whether the current mutation in these variants provides functional adaptation.

In conclusion, our study establishes a basis for tracking the evolutionary dynamics of ToBRFV in Morocco. Future work is needed to characterize the biological relevance of the observed pattern among the Moroccan isolates with respect to host responses.

## Data Availability

ToBRFV Tom1 (PZ370457), ToBRFV Tom2 (PZ370458), ToBRFV Tom3 (PZ370459), ToBRFV Tom5 (PZ370460), ToBRFV Tom6 (PZ370461), ToBRFV Tom7 (PZ370462), ToBRFV Tom9 (PZ370463), ToBRFV Tom10 (PZ370464), ToBRFV Tom11 (PZ370465), ToBRFV Tom12 (PZ370466), ToBRFV Tom13 (PZ370467), ToBRFV Tom15 (PZ370468), ToBRFV Tom16 (PZ370469), ToBRFV Tom18 (PZ370470), ToBRFV Tom19 (PZ370471), ToBRFV Tom20 (PZ370472), ToBRFV Tom21 (PZ370473), ToBRFV Tom23 (PZ370474), ToBRFV Tom25 (PZ370475), ToBRFV Tom27 (PZ370476), ToBRFV Tom28 (PZ370477), ToBRFV Tom30 (PZ370478), ToBRFV Tom31 (PZ370479), ToBRFV Tom34 (PZ370480), ToBRFV Tom36 (PZ370481), ToBRFV Tom37 (PZ370482), ToBRFV Tom38 (PZ370483), ToBRFV Tom39 (PZ370484), ToBRFV Tom40 (PZ370485), ToBRFV Tom42 (PZ370486), ToBRFV Tom49 (PZ370487), ToBRFV Tom50 (PZ370488), ToBRFV Tom51 (PZ370489), ToBRFV Tom55 (PZ370490), ToBRFV Tom56 (PZ370491), ToBRFV Tom67 (PZ370492), ToBRFV Tom90 (PZ370493), ToBRFV Tom92 (PZ370494).

## Supplementary Materials

The following supporting information can be downloaded at https://www.mdpi.com/article/10.3390/v18091032/s1. Figure S1: Nanopore sequencing output for the analyzed amplicons. (A) Total number of sequencing reads obtained for each sample. (B) Sequencing coverage depth obtained for each sample. Figure S2: Relationships among the tomato brown rugose fruit virus (ToBRFV) sequences from Morocco and the rest of the world using “median-joining” haplotype networks. ToBRFV sequences from Morocco are highlighted in dark green Table S1: Selective pressures acting on site-by-site codon on partial RNA-dependent RNA polymerase; Table S2: Selective pressures acting on site-by-site codon on partial movement protein gene; Table S3: Selective pressures acting on site-by-site codon on coat protein gene.

## Abbreviations

The following abbreviations are used in this manuscript:

CP	Coat protein
HN	Haplotype network
MP	Movement protein
NCBI	National Center for Biotechnology Information
NL	The Netherlands
RdRp	RNA-dependent RNA polymerase
ToBRFV	Tomato brown rugose fruit virus
UK	The United Kingdom
