# Supplementary material for: Geographic Structuring of the Tomato Brown Rugose Fruit Virus Populations in Morocco
Source: Viruses. 2026 Sep 17;18(9):1032. doi: 10.3390/v18091032 (PMC13611845; doi:10.3390/v18091032)
Supplement: Supplementary file 1 [file viruses-18-01032-s001.zip › supplementary_data.pdf]

**Supplementary data**

**Table S1.** Selective pressures acting on site-by-site codon on the partial RNA-dependent RNA polymerase gene.

**Table S2.** Selective pressures acting on site-by-site codon on the movement of protein gene.

**Table S3.** Selective pressures acting on site-by-site codon on the capsid protein gene.

**Figure S1.** Nanopore sequencing output for the analyzed amplicons. (A) Total number of sequencing reads for each sample. (B) Sequencing coverage depth obtained from each sample.

**Figure S2.** Relationships among the tomato brown rugose fruit virus (ToBRFV) sequences from Morocco and the world using "Median-joining" haplotype network. ToBRFV sequences from Morocco are highlighted in dark green.
