# Supplementary material for: Geographic Structuring of the Tomato Brown Rugose Fruit Virus Populations in Morocco
Source: Viruses. 2026 Sep 17;18(9):1032. doi: 10.3390/v18091032 (PMC13611845; doi:10.3390/v18091032)
Supplement: Supplementary file 1 [file viruses-18-01032-s001.zip › Supplementary_figures.pdf]

Figure S1

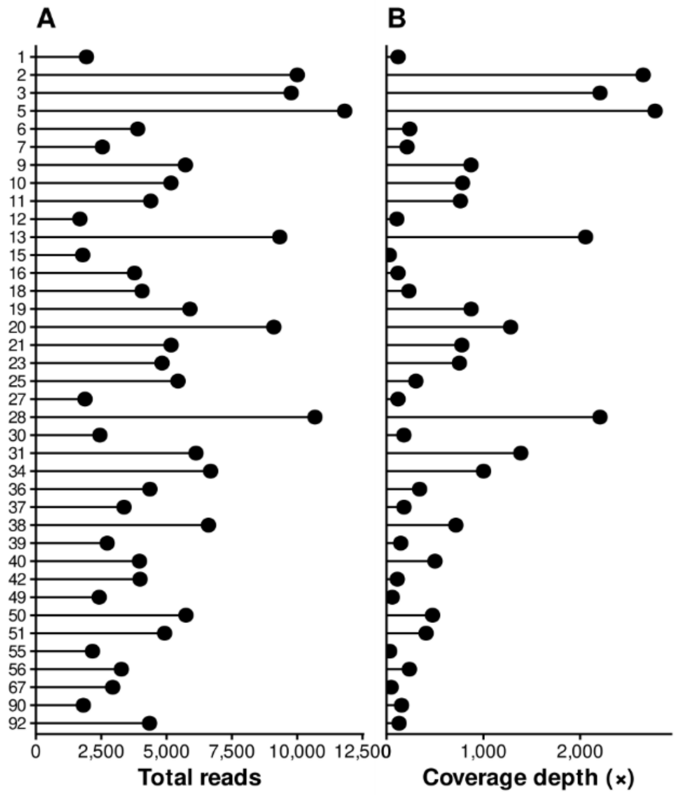

Figure S1. Nanopore sequencing output for the analyzed amplicons. (A) Total number of sequencing reads for each sample. (B) Sequencing coverage depth obtained from each sample.

Figure S2

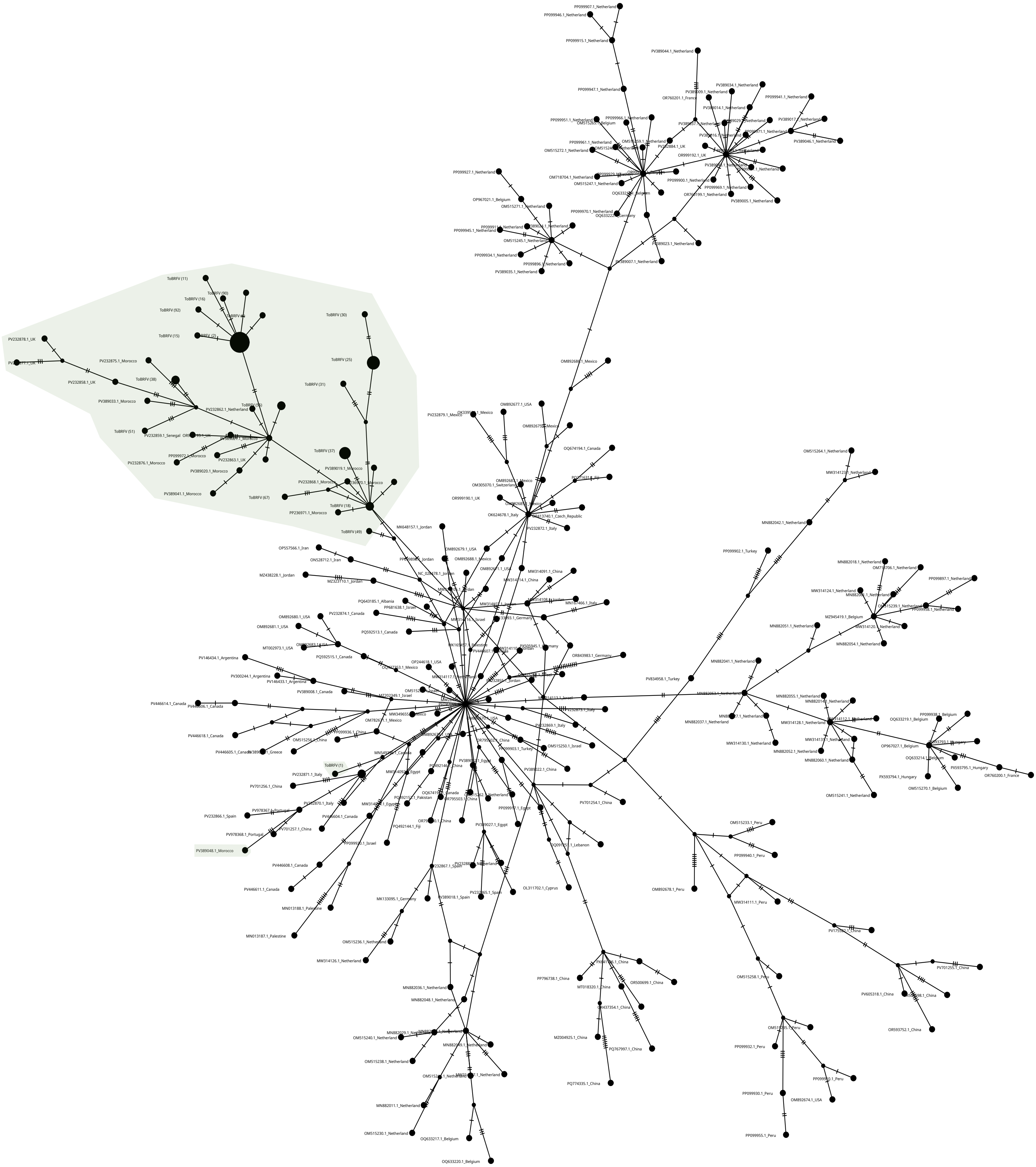

Figure S2. Relationships among the tomato brown rugose fruit virus (ToBRFV) sequences from Morocco and the world using “Median-joining” haplotype network. ToBRFV sequences from Morocco are highlighted in dark green.
